# (*E*)-2-[(*E*)-3-(Hy­droxy­imino)­butan-2-yl­idene]-*N*-methyl­hydrazinecarbothio­amide

**DOI:** 10.1107/S1600536811053621

**Published:** 2011-12-21

**Authors:** Halema Shaban Abduelftah, Amna Qasem Ali, Naser Eltaher Eltayeb, Siang Guan Teoh, Hoong-Kun Fun

**Affiliations:** a School of Chemical Sciences, Universiti Sains Malaysia, Minden, Penang, Malaysia; b Faculty of Science, Sabha University, Libya; c Department of Chemistry, International University of Africa, Khartoum, Sudan; d X-ray Crystallography Unit, School of Physics,Universiti Sains Malaysia, 11800 USM, Penang, Malaysia

## Abstract

In the title compound, C_6_H_12_N_4_OS, an intra­molecular N—H⋯N hydrogen-bond is present giving rise to an *S*(5) ring motif. In the crystal, double-stranded chains propagating along [10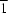] are formed *via* pairs of O—H⋯S and N—H⋯S hydrogen bonds. The chains are further stabilized by C—H⋯S interactions.

## Related literature

For standard bond lengths, see: Allen *et al.* (1987 ▶). For graph-set analysis of hydrogen bonds, see: Bernstein *et al.* (1995 ▶). For related structures, see: Choi *et al.* (2008 ▶). For the biological activity and pharmacological properties of thio­semi­carb­azones and their metal complexes, see: Cowley *et al.* (2002 ▶); Ming (2003 ▶); Lobana *et al.* (2004 ▶, 2007 ▶).
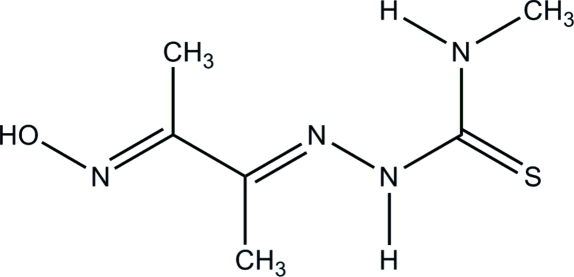


## Experimental

### 

#### Crystal data

C_6_H_12_N_4_OS
*M* *_r_* = 188.26
Triclinic, 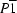

*a* = 5.5205 (1) Å
*b* = 8.6077 (2) Å
*c* = 9.5650 (2) Å
α = 79.750 (1)°
β = 89.509 (1)°
γ = 85.083 (1)°
*V* = 445.61 (2) Å^3^
*Z* = 2
Mo *K*α radiation
μ = 0.32 mm^−1^
*T* = 100 K
0.51 × 0.25 × 0.07 mm

#### Data collection

Bruker APEXII CCD diffractometer
Absorption correction: multi-scan (*SADABS*; Bruker, 2005 ▶) *T*_min_ = 0.854, *T*_max_ = 0.978
12035 measured reflections
3256 independent reflections
2920 reflections with *I* > 2σ(*I*)
*R*_int_ = 0.020

#### Refinement

*R*[*F* ^2^ > 2σ(*F* ^2^)] = 0.031
*wR*(*F* ^2^) = 0.081
*S* = 1.08
3256 reflections
124 parameters
H atoms treated by a mixture of independent and constrained refinement
Δρ_max_ = 0.40 e Å^−3^
Δρ_min_ = −0.39 e Å^−3^

### 

Data collection: *APEX2* (Bruker, 2005 ▶); cell refinement: *SAINT* (Bruker, 2005 ▶); data reduction: *SAINT*; program(s) used to solve structure: *SHELXTL* (Sheldrick, 2008 ▶); program(s) used to refine structure: *SHELXTL*; molecular graphics: *SHELXTL*; software used to prepare material for publication: *SHELXTL* and *PLATON* (Spek, 2009 ▶).

## Supplementary Material

Crystal structure: contains datablock(s) I, global. DOI: 10.1107/S1600536811053621/mw2038sup1.cif

Structure factors: contains datablock(s) I. DOI: 10.1107/S1600536811053621/mw2038Isup2.hkl

Supplementary material file. DOI: 10.1107/S1600536811053621/mw2038Isup3.cml

Additional supplementary materials:  crystallographic information; 3D view; checkCIF report

## Figures and Tables

**Table 1:** Hydrogen-bond geometry (Å, °)

*D*—H⋯*A*	*D*—H	H⋯*A*	*D*⋯*A*	*D*—H⋯*A*
N3—H1*N*3⋯S1^i^	0.877 (16)	2.781 (16)	3.6519 (9)	172.0 (14)
N4—H1*N*4⋯N2	0.848 (16)	2.155 (16)	2.5932 (11)	111.9 (13)
O1—H1*O*1⋯S1^ii^	0.857 (19)	2.437 (19)	3.2930 (8)	178.3 (17)
C4—H4*A*⋯S1^i^	0.98	2.69	3.3991 (12)	129

Symmetry codes: (i) 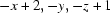; (ii) 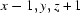; (iii) 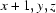; (iv) 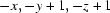.

**Fig. 1.**
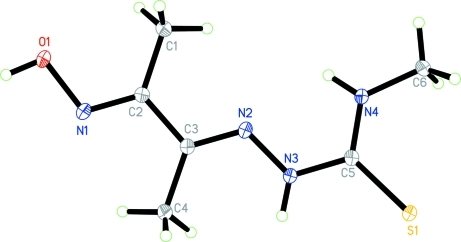
The molecular structure of the title compound with 50% probability displacement ellipsoids and the atom-numbering scheme.

**Fig. 2.**
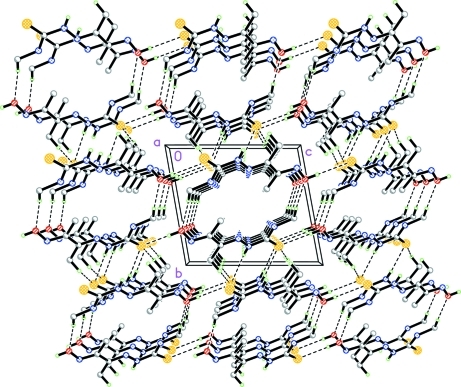
The crystal packing of the title compound viewed down the a axis. Hydrogen bonds are shown as dashed lines.

## supplementary crystallographic information

### Comment

Thiosemicarbazones and their metal complexes have attracted significant attention because of their wide-ranging biological and pharmacological activities related to specific structures as well as chemical properties (Cowley *et al.*, 2002; Ming, 2003; Lobana *et al.*, 2007; Lobana *et al.*, 2004). In this paper we report the crystal structure of (*E*)-2-((*E*)-3-(hydroxyimino)butan-2-ylidene)-*N*-methylhydrazinecarbothioamide (Fig. 1).

In the title compound, C_6_H_12_N_4_OS, butane is the longest carbon-carbon chain with the oxime group bound to C2 and the 4-methyl-3-thiosemicarbazide moiety bound to C3. The two methyl groups C1 and C4 are *trans* to each other. The torsion angles of the chains (O1/N1/C2/C3), (C1/C2/C3/C4) and (N2/N3/C5/N4) are 178.35 (8)°, -176.26 (10)° and -5.71 (13)°, respectively, indicating the near-planarity of the molecular backbone. All bond lengths and angles are normal (Allen *et al.*, 1987).

Cyclic intramolecular  N4—H1N4···N2, C1—H1B···N2 and C4—H4B···N1 hydrogen-bonding interactions [graph set *S*(5), (Bernstein *et al.*, 1995)] are present (Table 1) with the latter two being notably weaker than the first. In the crystal molecules are connected through intermolecular O1—H1O1···S1 hydrogen bonds into infinite one-dimensional chains which propagate along [1 0 -1]. In addition, intermolecular N3—H1N3···S1, C4—H4A···S1 and C6—H6A···O1 hydrogen bonds associate these chains into sheets while the sheets are tied together *via* C4—H4C···O1 interactions (Fig. 2, Table 1). As a consequence of the C4—H4A···S1 and C4—H4C···O1 interactions, a rather short H4B-H4B contact is forced between adjacent molecules in the sheet.

### Experimental

To a hot stirred solution of 2,3-butanedione monoxime (1.01 g, 10 mmole) in ethanol (20 ml) containing a few drops of glacial acetic acid was added 4-methyl-3-thiosemicarbazide (1.05 g, 10 mmole) dissolved in ethanol (20 ml). The reaction mixture was then heated under reflux for 3 h. The mixture was filtered and left to cool, the resulting white solid was collected by suction filtration and washed with cold EtOH. The white crystals were grown from ethanol soultion by slow evaporation at room temperature, yield, 78.8%, m.p., 487.5–490 K.

### Refinement

N and O bound H atoms were located in a difference Fourier map and were refined freely. The remaining H atoms were positioned geometrically and refined using a riding model with C—H = 0.98 and *U*_iso_(H) = 1.5*U*_eq_(C) for methyl groups. The highest residual electron density peak is located 0.63 Å from C2 and the deepest hole is located 0.68 Å from C4.

### Crystal data

**Table:**

C_6_H_12_N_4_OS	*Z* = 2
*M**_r_* = 188.26	*F*(000) = 200
Triclinic, *P*1	*D*_x_ = 1.403 Mg m^−^^3^
Hall symbol: -P 1	Melting point = 487.5–490 K
*a* = 5.5205 (1) Å	Mo *K*α radiation, λ = 0.71073 Å
*b* = 8.6077 (2) Å	Cell parameters from 7387 reflections
*c* = 9.5650 (2) Å	θ = 2.4–32.7°
α = 79.750 (1)°	µ = 0.32 mm^−^^1^
β = 89.509 (1)°	*T* = 100 K
γ = 85.083 (1)°	Plate, colourless
*V* = 445.61 (2) Å^3^	0.51 × 0.25 × 0.07 mm

### Data collection

**Table:**

Bruker APEXII CCD diffractometer	3256 independent reflections
Radiation source: fine-focus sealed tube	2920 reflections with *I* > 2σ(*I*)
graphite	*R*_int_ = 0.020
φ and ω scans	θ_max_ = 32.7°, θ_min_ = 2.2°
Absorption correction: multi-scan (*SADABS*; Bruker, 2005)	*h* = −8→8
*T*_min_ = 0.854, *T*_max_ = 0.978	*k* = −13→13
12035 measured reflections	*l* = −14→14

### Refinement

**Table:**

Refinement on *F*^2^	Primary atom site location: structure-invariant direct methods
Least-squares matrix: full	Secondary atom site location: difference Fourier map
*R*[*F*^2^ > 2σ(*F*^2^)] = 0.031	Hydrogen site location: inferred from neighbouring sites
*wR*(*F*^2^) = 0.081	H atoms treated by a mixture of independent and constrained refinement
*S* = 1.08	*w* = 1/[σ^2^(*F*_o_^2^) + (0.0347*P*)^2^ + 0.1679*P*] where *P* = (*F*_o_^2^ + 2*F*_c_^2^)/3
3256 reflections	(Δ/σ)_max_ = 0.001
124 parameters	Δρ_max_ = 0.40 e Å^−^^3^
0 restraints	Δρ_min_ = −0.39 e Å^−^^3^

### Special details

**Table:**

Geometry. All e.s.d.'s (except the e.s.d. in the dihedral angle between two l.s. planes) are estimated using the full covariance matrix. The cell e.s.d.'s are taken into account individually in the estimation of e.s.d.'s in distances, angles and torsion angles; correlations between e.s.d.'s in cell parameters are only used when they are defined by crystal symmetry. An approximate (isotropic) treatment of cell e.s.d.'s is used for estimating e.s.d.'s involving l.s. planes.
Refinement. Refinement of *F*^2^ against ALL reflections. The weighted *R*-factor *wR* and goodness of fit *S* are based on *F*^2^, conventional *R*-factors *R* are based on *F*, with *F* set to zero for negative *F*^2^. The threshold expression of *F*^2^ > σ(*F*^2^) is used only for calculating *R*-factors(gt) *etc*. and is not relevant to the choice of reflections for refinement. *R*-factors based on *F*^2^ are statistically about twice as large as those based on *F*, and *R*- factors based on ALL data will be even larger.

### Fractional atomic coordinates and isotropic or equivalent isotropic displacement parameters (Å^2^)

**Table:**

	*x*	*y*	*z*	*U*_iso_*/*U*_eq_	
S1	0.94334 (4)	0.16073 (3)	0.30007 (2)	0.01590 (7)	
O1	−0.03857 (14)	0.29827 (9)	0.95645 (8)	0.02032 (15)	
N1	0.16712 (15)	0.21630 (10)	0.90822 (9)	0.01569 (15)	
N2	0.44402 (14)	0.22893 (9)	0.57941 (8)	0.01351 (14)	
N3	0.64505 (15)	0.16555 (10)	0.51705 (8)	0.01452 (15)	
N4	0.51169 (15)	0.32555 (10)	0.31045 (8)	0.01554 (15)	
C1	0.02246 (18)	0.37515 (12)	0.67902 (10)	0.01835 (18)	
H1A	−0.1458	0.3575	0.7064	0.028*	
H1B	0.0464	0.3628	0.5798	0.028*	
H1C	0.0556	0.4825	0.6895	0.028*	
C2	0.19230 (17)	0.25693 (11)	0.77265 (10)	0.01371 (16)	
C3	0.40835 (17)	0.17983 (11)	0.71298 (10)	0.01444 (16)	
C4	0.5672 (2)	0.05499 (14)	0.80647 (11)	0.0258 (2)	
H4A	0.6119	−0.0328	0.7560	0.039*	
H4B	0.4791	0.0158	0.8932	0.039*	
H4C	0.7146	0.1001	0.8314	0.039*	
C5	0.68449 (17)	0.22272 (10)	0.37711 (9)	0.01296 (15)	
C6	0.51377 (18)	0.39340 (12)	0.15976 (10)	0.01718 (18)	
H6A	0.3703	0.4686	0.1362	0.026*	
H6B	0.5111	0.3086	0.1038	0.026*	
H6C	0.6612	0.4486	0.1379	0.026*	
H1N3	0.754 (3)	0.0948 (18)	0.5626 (16)	0.025 (4)*	
H1N4	0.387 (3)	0.3399 (18)	0.3605 (17)	0.026 (4)*	
H1O1	−0.040 (3)	0.263 (2)	1.046 (2)	0.039 (4)*	

### Atomic displacement parameters (Å^2^)

**Table:**

	*U*^11^	*U*^22^	*U*^33^	*U*^12^	*U*^13^	*U*^23^
S1	0.01340 (11)	0.02020 (11)	0.01157 (10)	0.00444 (8)	0.00346 (7)	0.00102 (7)
O1	0.0191 (3)	0.0277 (4)	0.0116 (3)	0.0092 (3)	0.0044 (3)	−0.0022 (3)
N1	0.0146 (4)	0.0190 (3)	0.0124 (3)	0.0039 (3)	0.0031 (3)	−0.0025 (3)
N2	0.0128 (3)	0.0160 (3)	0.0112 (3)	0.0009 (3)	0.0029 (3)	−0.0020 (3)
N3	0.0135 (3)	0.0179 (3)	0.0102 (3)	0.0041 (3)	0.0024 (3)	0.0002 (3)
N4	0.0135 (4)	0.0203 (4)	0.0103 (3)	0.0044 (3)	0.0026 (3)	0.0012 (3)
C1	0.0175 (4)	0.0223 (4)	0.0126 (4)	0.0057 (3)	0.0007 (3)	0.0005 (3)
C2	0.0134 (4)	0.0154 (4)	0.0113 (4)	0.0017 (3)	0.0009 (3)	−0.0013 (3)
C3	0.0151 (4)	0.0161 (4)	0.0107 (4)	0.0028 (3)	0.0019 (3)	−0.0006 (3)
C4	0.0265 (5)	0.0314 (5)	0.0129 (4)	0.0159 (4)	0.0054 (4)	0.0048 (4)
C5	0.0129 (4)	0.0145 (4)	0.0108 (4)	0.0004 (3)	0.0014 (3)	−0.0014 (3)
C6	0.0180 (4)	0.0202 (4)	0.0106 (4)	0.0046 (3)	0.0013 (3)	0.0017 (3)

### Geometric parameters (Å, °)

**Table:**

S1—C5	1.6914 (9)	C1—H1A	0.9800
O1—N1	1.4034 (10)	C1—H1B	0.9800
O1—H1O1	0.858 (19)	C1—H1C	0.9800
N1—C2	1.2911 (12)	C2—C3	1.4752 (13)
N2—C3	1.2912 (12)	C3—C4	1.4944 (13)
N2—N3	1.3733 (11)	C4—H4A	0.9800
N3—C5	1.3639 (12)	C4—H4B	0.9800
N3—H1N3	0.876 (15)	C4—H4C	0.9800
N4—C5	1.3285 (12)	C6—H6A	0.9800
N4—C6	1.4560 (12)	C6—H6B	0.9800
N4—H1N4	0.847 (16)	C6—H6C	0.9800
C1—C2	1.4977 (13)		
			
N1—O1—H1O1	104.1 (12)	N2—C3—C2	115.15 (8)
C2—N1—O1	111.22 (8)	N2—C3—C4	125.00 (9)
C3—N2—N3	118.54 (8)	C2—C3—C4	119.84 (8)
C5—N3—N2	117.75 (8)	C3—C4—H4A	109.5
C5—N3—H1N3	118.0 (10)	C3—C4—H4B	109.5
N2—N3—H1N3	124.1 (10)	H4A—C4—H4B	109.5
C5—N4—C6	124.53 (8)	C3—C4—H4C	109.5
C5—N4—H1N4	114.1 (11)	H4A—C4—H4C	109.5
C6—N4—H1N4	120.9 (11)	H4B—C4—H4C	109.5
C2—C1—H1A	109.5	N4—C5—N3	116.23 (8)
C2—C1—H1B	109.5	N4—C5—S1	124.44 (7)
H1A—C1—H1B	109.5	N3—C5—S1	119.33 (7)
C2—C1—H1C	109.5	N4—C6—H6A	109.5
H1A—C1—H1C	109.5	N4—C6—H6B	109.5
H1B—C1—H1C	109.5	H6A—C6—H6B	109.5
N1—C2—C3	115.02 (8)	N4—C6—H6C	109.5
N1—C2—C1	124.30 (8)	H6A—C6—H6C	109.5
C3—C2—C1	120.68 (8)	H6B—C6—H6C	109.5
			
C3—N2—N3—C5	−177.76 (8)	N1—C2—C3—C4	4.82 (14)
O1—N1—C2—C3	178.35 (8)	C1—C2—C3—C4	−176.26 (10)
O1—N1—C2—C1	−0.52 (13)	C6—N4—C5—N3	−176.61 (9)
N3—N2—C3—C2	178.38 (8)	C6—N4—C5—S1	3.43 (14)
N3—N2—C3—C4	−1.47 (15)	N2—N3—C5—N4	−5.71 (13)
N1—C2—C3—N2	−175.03 (9)	N2—N3—C5—S1	174.25 (6)
C1—C2—C3—N2	3.88 (13)		

### Hydrogen-bond geometry (Å, °)

**Table:**

*D*—H···*A*	*D*—H	H···*A*	*D*···*A*	*D*—H···*A*
N3—H1N3···S1^i^	0.877 (16)	2.781 (16)	3.6519 (9)	172.0 (14)
N4—H1N4···N2	0.848 (16)	2.155 (16)	2.5932 (11)	111.9 (13)
O1—H1O1···S1^ii^	0.857 (19)	2.437 (19)	3.2930 (8)	178.3 (17)
C1—H1B···N2	0.98	2.39	2.7919 (13)	104
C4—H4A···S1^i^	0.98	2.69	3.3991 (12)	129
C4—H4B···N1	0.98	2.35	2.7698 (14)	105
C4—H4C···O1^iii^	0.98	2.71	3.6173 (16)	154
C6—H6A···O1^iv^	0.98	2.63	3.6011 (12)	170

Symmetry codes: (i) −*x*+2, −*y*, −*z*+1; (ii) *x*−1, *y*, *z*+1; (iii) *x*+1, *y*, *z*; (iv) −*x*, −*y*+1, −*z*+1.

## References

[bb1] Allen, F. H., Kennard, O., Watson, D. G., Brammer, L., Orpen, A. G. & Taylor, R. (1987). *J. Chem. Soc. Perkin Trans. 2*, pp. S1–19.

[bb2] Bernstein, J., Davis, R. E., Shimoni, L. & Chang, N.-L. (1995). *Angew. Chem. Int. Ed. Engl.* **34**, 1555–1573.

[bb3] Bruker (2005). *APEX2*, *SAINT* and *SADABS* Bruker AXS Inc., Madison, Wisconsin, USA.

[bb4] Choi, K.-Y., Yang, S.-M., Lee, K.-C., Ryu, H., Lee, C. H., Seo, J. & Suh, M. (2008). *Transition Met. Chem.* **33**, 99–105.

[bb5] Cowley, A. R., Dilworth, J. R., Donnelly, P. S., Labisbal, E. & Sousa, A. (2002). *J. Am. Chem. Soc.* **124**, 5270–5271. 10.1021/ja012668z 11996559

[bb6] Lobana T. S., Rekha & Butcher, R. J. (2004). *Transition Met. Chem.* **29**, 291–295.

[bb7] Lobana T. S., Rekha, Pannu A.P.S., Hundal G., Butcher R. J. & Castineiras A. (2007). *Polyhedron*, **26**, 2621–2628*.*

[bb8] Ming, L.-J. (2003). *Med. Res. Rev.* **23**, 697–762. 10.1002/med.10052 12939790

[bb9] Sheldrick, G. M. (2008). *Acta Cryst.* A**64**, 112–122. 10.1107/S0108767307043930 18156677

[bb10] Spek, A. L. (2009). *Acta Cryst.* D**65**, 148–155. 10.1107/S090744490804362X PMC2631630 19171970

